# The fibro-adipogenic progenitor APOD+DCN+LUM+ cell population in aggressive carcinomas

**DOI:** 10.1007/s10555-024-10181-y

**Published:** 2024-03-11

**Authors:** Lingyi Cai, Mikhail G. Kolonin, Dimitris Anastassiou

**Affiliations:** 1 Department of Systems Biology, Columbia University, New York, NY USA; 2 Department of Electrical Engineering, Columbia University, New York, NY USA; 3 The Brown Foundation Institute of Molecular Medicine for the Prevention of Human Diseases, The University of Texas Health Sciences Center at Houston, Houston, TX USA; 4 Herbert Irving Comprehensive Cancer Center, Columbia University, New York, NY USA

## Abstract

We identified a progenitor cell population highly enriched in samples from invasive and chemo-resistant carcinomas, characterized by a well-defined multigene signature including APOD, DCN, and LUM. This cell population has previously been labeled as consisting of inflammatory cancer-associated fibroblasts (iCAFs). The same signature characterizes naturally occurring fibro-adipogenic progenitors (FAPs) as well as stromal cells abundant in normal adipose tissue. Our analysis of human gene expression databases provides evidence that adipose stromal cells (ASCs) are recruited by tumors and undergo differentiation into CAFs during cancer progression to invasive and chemotherapy-resistant stages.

**Supplementary Information:**

The online version contains supplementary material available at 10.1007/s10555-024-10181-y.

During carcinoma progression, mesenchymal stromal cells become recruited to tumors and contribute to the pool of CAFs. The heterogeneity of CAF populations, changing during disease progression, has been recognized [1]. CAFs can be derived from tissue-resident fibroblasts as well as various other lineages. However, CAF sub-populations are diverse and have incompletely understood effects on disease progression and resistance to therapy. Here, by analyzing public databases of human single-cell RNAseq (scRNA-seq) data, we have identified a CAF progenitor population marked by expression of genes *APOD, DCN, LUM*, typically accompanied by expression of *CFD, CXCL14, PTGDS, MGP, SERPINF1*, and *DPT*. We show that these cells are prominent in the following two settings:

1. Naturally occurring in cancer-free individuals as (a) ASCs previously identified as adipocyte progenitors enriched in the stromal vascular fraction (SVF) of adipose tissue [2], as well as (b) fibro-adipogenic progenitors (FAPs) in skeletal muscle [3]. We refer to the corresponding gene co-expression signature as “the ASC/FAP signature.”
2. Enriched in the tumor microenvironment of invasive and chemoresistant carcinomas of various types.

The ASC/FAP progenitor population has both adipogenic and fibroblastic differentiation potential. In this work, we provide evidence that it is recruited by cancer cells and undergoes differentiation into CAFs (Fig. 1a).

**Fig. 1:**
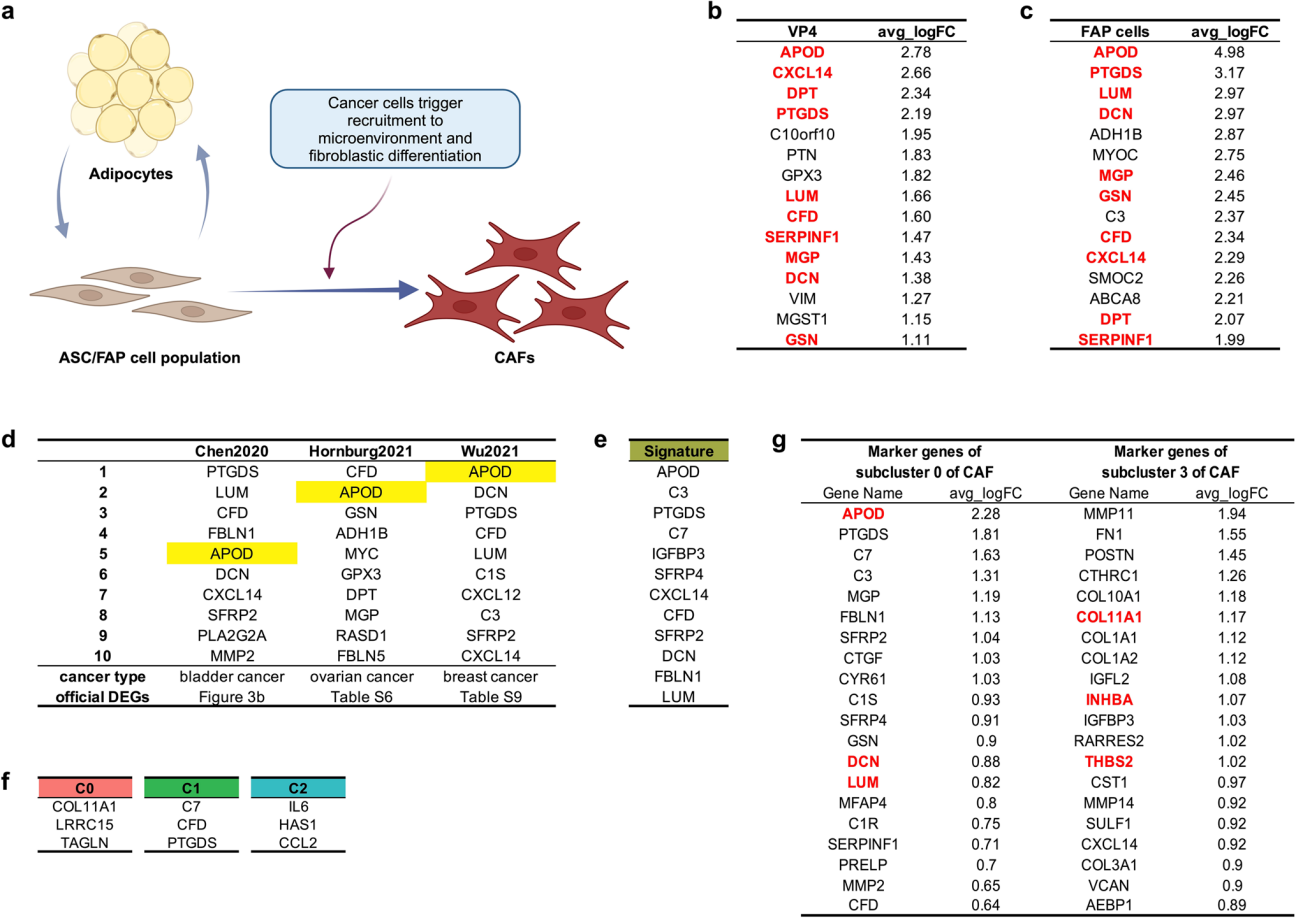
The ASC/FAP population and its marker genes. **a** Model indicating the underlying differentiation mechanisms. Created with BioRender.com. **b** Top DEGs of the adipose stromal cell (ASC) cluster VP4 from normal adipose tissue (from ref. [2]). **c** Top DEGs of cluster fibro-adipogenic progenitors (FAP) (from ref. [3]). Each of the ten genes in red font appears in both lists (*P* = 10^-30^ by hypergeometric test). **d** Top ten genes from lists of DEGs provided in [6–8] of clusters containing the ASC/FAP population in bladder, ovarian, and breast cancer, ranked by average fold change. **e** PDAC “chemo-resistance signature.” **f** Representative genes of three fibroblastic subpopulations in Fig. 5D of ref. [14] C0 is adjacent to C1, while IL6+ iCAFs form a distinct cluster, C2. **g** Marker genes of adjacent clusters C0 and C3 in ref. [19]. Key marker genes of the two populations are in red font

The ASC/FAP signature reflects remarkably similar lists of marker genes in the ASC cluster [2] (Fig. 1b) and the FAP cluster [3] (Fig. 1c). The presence of the same signature in both ASCs and FAPs is also consistent with the finding that adipose tissue is a source of FAP-like cells recruited to skeletal muscle undergoing remodeling [4]. To demonstrate that the ASC/FAP population exists in abundance in each of the 25 adipose tissue samples presented previously [2], we analyzed each of those samples derived from 14 cancer-free individuals. We used an attractor algorithm [5] (Methods in Supplementary Information) designed to converge to a ranked list of genes identifying the core co-expression characterizing cell populations. We independently generated the lists of top-ranked genes for each sample. Supplementary Table 1 shows that all genes mentioned above are consistently top ranked, while Supplementary Fig. 1 demonstrates the abundance of the ASC/FAP population in the SVF.

We identified similarly enriched ASC/FAP genes in cell clusters of bladder [6], ovarian [7], and breast [8] cancer (Fig. 1d). The twelve top differentially expressed genes (DEGs) of a population were also found to have a three-fold enrichment in chemo-resistant samples of pancreatic cancer, referred to as constituting a “chemo-resistance signature” as shown in their extended data Fig. 7b (Fig.1e) [9]. The strong enrichment of the ASC/FAP cell population in multiple cancer types is consistent with the recruitment of this particular progenitor population in aggressive and chemo-resistant cancers. Consistently, lineage tracing and transplantation studies in mouse models indicate that ASCs can be recruited by carcinomas to promote cancer progression [10, 11].

The recruited ASC/FAP cells are typically misrepresented as inflammatory CAFs (“iCAFs”) because they are often included in nonhomogeneous computationally derived clusters that also contain such cells. iCAFs have been defined as fibroblasts expressing *IL6* and additional inflammatory mediators [12], and the expression of *IL6* has remained a requirement in a Consensus Statement of experts describing iCAFs [13]. *IL6* expression can also be induced in a subset of cells in such clusters. However, the ASC/FAP population in its original non-inflammatory status should not be confused with IL6+ iCAFs. For example, the three representative genes for each fibroblastic subpopulation in Fig. 5D of Dominguez et al. [14] are shown in Fig. 1f. Cluster 1 is marked by genes *C7*, *CFD* and *PTGDS*, all three of which are among the twelve in the chemo-resistance ASC/FAP signature in Fig. 1e. Cluster 2 is marked by *IL6*, *HAS1* and *CCL2*, all among the iCAF signature genes defined previously [15], hence identifying it as the true iCAF population. It was recently suggested that there are “two separate populations of iCAFs: one IL6 positive and another IL6 negative.” [16] In fact, the IL6-negative cluster largely contains the APOD+ ASC/FAP population and should not be characterized as iCAFs.

Single-cell analysis revealed that, in aggressive cancers, cells with the ASC/FAP signature convert to a particular type of CAFs expressing *COL11A1*, *THBS2*, and *INHBA* [17]. This CAF signature was first reported by Kim et al. [18] and included additional genes such as *POSTN*, *COL10A1*, and *MMP11*. One example consistent with the transition is described by Fig. 2a of Wang et al. [19], in which cluster C0 expresses *APOD*, *DCN*, and *LUM*, while cluster C3 adjacent to it expresses *COL11A1*, *THBS2*, and *INHBA* (Fig. 1g). Furthermore, the presence of the COL11A1+ cluster 0, adjacent to C7+CFD+PTGDS+ cluster 1 in Fig. 5D from Dominguez et al., [14] is also consistent with the transition, as is the presence of gene *POSTN* together with *APOD*, *CFD*, and *CXCL14* in the same “poor prognosis” cluster (CAF_0) [20] in gastric cancer. *COL11A1* is also identified as the collagen marker most strongly associated with poor prognosis [16].

In summary, this commentary draws attention to the APOD+DCN+LUM+ gene signature as representing an important unrecognized population in cancer. These cells are likely derived from ASC/FAPs recruited by carcinomas; however, their origination from other sources cannot be excluded. Their differentiation into COL11A1+ CAFs, accompanying the transition to metastasis, may underlie a mechanism that accounts for the role of adipose tissue in cancer aggressiveness [21]. Genetic manipulations of ASCs in cell culture and animal models of carcinoma progression to chemo-resistance will be necessary to obtain further evidence for the origin of CAFs associated with poor prognosis of cancer. Further research may help in developing appropriate therapeutics targeting the underlying mechanisms.

### Supplementary information

ESM 1 (DOCX 416 KB)

## Data Availability

The scRNA-seq data used for the presented results were downloaded from Gene Expression Omnibus (GEO) with accession number GSE129363. The algorithm we developed and used is available at https://github.com/LingyiC/adaptiveAttractor.
