# Supplementary material for: The fibro-adipogenic progenitor APOD+DCN+LUM+ cell population in aggressive carcinomas
Source: Cancer Metastasis Rev. 2024 Mar 11;43(3):977–80. doi: 10.1007/s10555-024-10181-y (PMC11300568; doi:10.1007/s10555-024-10181-y)
Supplement: Supplementary file 1 — ESM 1 (DOCX 416 KB) [file 10555_2024_10181_MOESM1_ESM.docx]

**Supplementary Information**

**The signature of the main stromal cell population in the SVF of adipose tissue.**

We applied the adaptive attractor algorithm (Methods) separately and independently in each of the 25 adipose tissue samples of ref.^1^. It is an iterative algorithm designed to converge to a ranked list of genes at the core of co-expression based on a seed gene, in this case the fibroblastic marker LUM. The converged results of the algorithm are identical using as seed gene any among the marker genes mentioned in the main text. Supplementary Table 1 shows the ranked lists demonstrating remarkable similarity (main text). Furthermore, the number of the APOD+DCN+LUM+ cells is large. For example, referring to the richest sample SVF2-SAT: It has totally 5,846 cells, out of which there are 2,884 cells expressing both LUM and DCN, each at normalized values greater than 2. Among those 2,884 stromal cells, 2,460 (85%) also express APOD at a normalized value greater than 2. Supplementary Fig. 1 shows feature plots for that sample demonstrating the co-expression and the abundance of the ASC/FAP population.

# **Methods**

## **Adaptive attractor algorithm**

Detailed descriptions of the algorithm can be found in the original paper^2^ introducing the attractor algorithm and examples of analysis of single-cell data analysis can be found in ref.^3^. In this paper, we are using an adaptive version of the algorithm, in which the value of the exponent parameter is optimized to identify strongest co-expression features. It is gradually decreased from a=5 to a=2 by default to maximize the strength of the 10th-ranked genes in the converged attractor.

# **Data availability**

The scRNA-seq data from 25 adipose samples were downloaded from Gene Expression Omnibus (GEO) with accession number GSE129363^1^. The count matrix was normalized using the Seurat R package (v4.1.0)^4^.

# **Code availability**

The code for adaptive attractor algorithm and reproducing Supplementary Table 1 is available at GitHub: <https://github.com/LingyiC/adaptiveAttractor>.

# **Supplementary Table and Figure**

## **Supplementary Table 1**

Top 40 genes of LUM-seeded adaptive attractors for 25 human adipose samples. Highlighted are marker genes APOD, DCN, LUM, CFD, CXCL14, PTGDS, MGP, SERPINF1, DPT and GSN.

## **Supplementary Figure 1**

t-SNE feature plots of sample SVF2-SAT^1^ demonstrating the abundance of the APOD+DCN+LUM+ cell population. Shown are several co-expressed genes of the ASC signature as well as markers for immune and endothelial cells demonstrating expression the remaining samples.


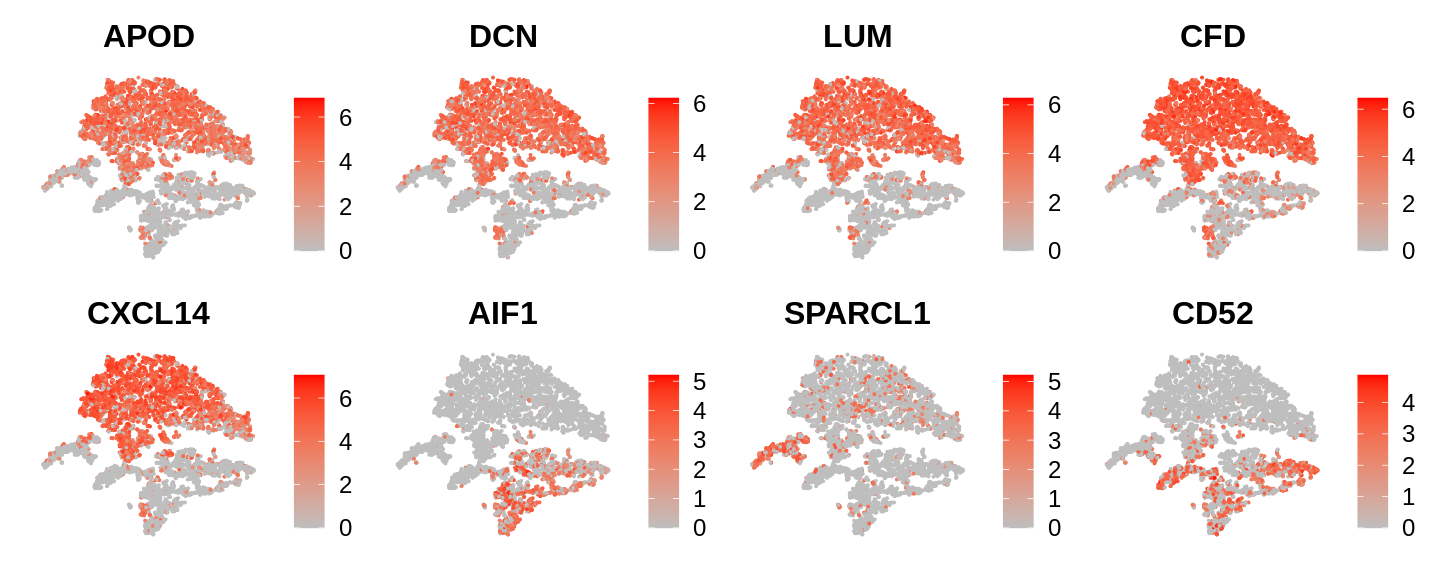


# **References**

1. Vijay, J. *et al.* Single-cell analysis of human adipose tissue identifies depot- and disease-specific cell types. *Nat. Metab.* **2**, 97–109 (2020).

2. Cheng, W.-Y., Ou Yang, T.-H. & Anastassiou, D. Biomolecular Events in Cancer Revealed by Attractor Metagenes. *PLOS Comput. Biol.* **9**, e1002920 (2013).

3. Zhu, K., Cai, L., Cui, C., Toyos, J. R. de los & Anastassiou, D. Single-cell analysis reveals the pan-cancer invasiveness-associated transition of adipose-derived stromal cells into COL11A1-expressing cancer-associated fibroblasts. *PLOS Comput. Biol.* **17**, e1009228 (2021).

4. Hao, Y. *et al.* Integrated analysis of multimodal single-cell data. *Cell* **184**, 3573-3587.e29 (2021).
